# Tailoring a Combination Preerythrocytic Malaria Vaccine

**DOI:** 10.1128/IAI.01063-15

**Published:** 2016-02-24

**Authors:** Karolis Bauza, Erwan Atcheson, Tomas Malinauskas, Andrew M. Blagborough, Arturo Reyes-Sandoval

**Affiliations:** a The Jenner Institute, University of Oxford, Oxford, United Kingdom; b Division of Structural Biology, Wellcome Trust Centre for Human Genetics, University of Oxford, Oxford, United Kingdom; c Department of Life Sciences, Imperial College London, South Kensington, London, United Kingdom

## Abstract

The leading malaria vaccine candidate, RTS,S, based on the Plasmodium falciparum circumsporozoite protein (CSP), will likely be the first publicly adopted malaria vaccine. However, this and other subunit vaccines, such as virus-vectored thrombospondin-related adhesive protein (TRAP), provide only intermediate to low levels of protection. In this study, the Plasmodium berghei homologues of antigens CSP and TRAP are combined. TRAP is delivered using adenovirus- and vaccinia virus-based vectors in a prime-boost regime. Initially, CSP is also delivered using these viral vectors; however, a reduction of anti-CSP antibodies is seen when combined with virus-vectored TRAP, and the combination is no more protective than either subunit vaccine alone. Using an adenovirus-CSP prime, protein-CSP boost regime, however, increases anti-CSP antibody titers by an order of magnitude, which is maintained when combined with virus-vectored TRAP. This combination regime using protein CSP provided 100% protection in C57BL/6 mice compared to no protection using virus-vectored TRAP alone and 40% protection using adenovirus-CSP prime and protein-CSP boost alone. This suggests that a combination of CSP and TRAP subunit vaccines could enhance protection against malaria.

## INTRODUCTION

There are approximately 3.4 billion people at risk of malaria infection, 207 million cases and 627,000 deaths annually (1). An effective vaccine could have a greater impact than any other intervention (2, 3), and yet such a vaccine remains elusive.

Sterile protection against blood-stage malaria infection in both animal models and humans can be obtained by vaccination with whole radiation-attenuated sporozoites (spz) (4–6) or genetically attenuated parasites (7–11) incapable of developing beyond the liver stage. Difficulties associated with cost, production, and deployment of whole-parasite malaria vaccines to regions where malaria is endemic make it unlikely that such vaccines will play a central role in the control or eradication of malaria in the near future.

Subunit vaccines, consisting of single or multiple antigens from various stages of the malaria parasite, have been a focus of research development. These include the preerythrocytic-stage antigens circumsporozoite (CS) protein (12) and thrombospondin-related adhesive protein (TRAP) (13), the blood-stage antigens MSP-1 (14, 15), AMA-1 (16), and RH-5 (17), and the Plasmodium vivax antigen Duffy binding protein (18, 19); the transmission-blocking antigens Pfs25, Pvs25, Pfs230, and Pfs48/45 have also been investigated as potential subunit vaccines (20–23).

The current leading malaria vaccine candidate, RTS,S, is a subunit vaccine undergoing phase III clinical trials in Africa (12). This vaccine consists of part of the CS protein of Plasmodium falciparum malaria fused to the hepatitis B virus surface antigen (HBsAg) and coexpressed in yeast with HBsAg. The vaccine is administered as a protein-in-adjuvant formulation. The most recent results indicate that administering three doses of RTS,S protects 37% of infants (24) and 47% of children (12) against severe malaria.

Adenoviral-poxviral prime-boost protocols have been developed to maximize protective efficacy using viral-vectored vaccines (25). Viral vectored vaccines using chimpanzee adenoviral vector (ChAd63) or modified vaccinia strain Ankara (MVA) to deliver antigens show great promise, stimulating high T-cell responses (26–28). Multi-epitope TRAP (ME.TRAP) antigen delivered using virus-vectored vaccines produces very high levels of sterile protection in rodents (29), and in a recent phase IIa clinical trial (27) it was determined that this vaccine in a ChAd63-MVA prime-boost regime induced sterile protection in 21% of human volunteers.

With less than half of human volunteers seeing protective effects in recent trials, there is clearly a requirement for an improved, potent malaria vaccine. One potential improvement could be in combining two subunit vaccines to achieve enhanced protection. This is the approach explored here, using two of the leading malaria vaccine candidates, CSP and TRAP, and a commonly used murine model of malaria using Plasmodium berghei (30); murine models represent an inexpensive and useful way to examine vaccines in a preclinical setting before progression to human trials. CSP is involved in parasite motility and attachment and invasion of the liver of the vertebrate host (31). The first demonstration of anti-CSP antibody (Ab)-mediated protection was in P. berghei (32), and CD8^+^ T cells also play a role (33). TRAP also facilitates invasion of the liver (34, 35) and is involved in parasite motility (35, 36); P. berghei TRAP-specific CD8^+^ T cells have been shown to inhibit the liver stage (37).

Either CSP or TRAP used individually in a vaccine provides suboptimal levels of protection. In this study, their combination was tested and optimized.

## MATERIALS AND METHODS

### Protein expression and purification.

The mammalian codon optimized P. berghei CSP and TRAP genes were cloned into the pHLsec plasmid with the His tag at the 3′ end under the control of CMV enhancer and chick beta-actin promoter (38). DNA constructs were produced in Escherichia coli DH5α (Life Technologies) cells and purified using an endotoxin-free Plasmid Mega Kit (Qiagen). HEK-293T cells were transiently transfected using a DNA-polyethylenimine mix. This resulted in secreted proteins with N-terminal ETG and C-terminal GTK(His_6_) tags. The conditioned media with secreted proteins were dialyzed against phosphate-buffered saline (PBS), and proteins were purified by immobilized Co^2+^-affinity chromatography, followed by size exclusion chromatography in 20 mM Tris-HCl (pH 8.0)–300 mM NaCl. Protein size was verified by Western blotting with the PentaHis monoclonal primary antibody (1:1,000 dilution; Qiagen) and goat anti-mouse IgG peroxidase-conjugated secondary antibody (1:2,000; Sigma).

### Animals.

The age-matched, 6-week-old female inbred C57BL/6 (H-2b) and outbred CD1 (ICR) strains of mice used in this study were purchased from Harlan (USA). All animals and procedures were used in accordance with the terms of the UK Home Office Animals Act Project License. Procedures were approved by the University of Oxford Animal Care and Ethical Review Committee.

### Viral vector vaccines.

The *PbCSP* insert in viral vectored and protein vaccines was full length without modification (GenBank accession no. P23093.1). The *PbTRAP* insert (GenBank accession no. AAB63302.1) in viral vectors was modified by removal of the transmembrane domain, and a tPA (human plasminogen activator; GenBank accession no. K03021) leader sequence was inserted in the upstream region of the gene, replacing the native endogenous leader sequence, since it has been suggested that this adjustment facilitates antigen expression within the host cells (39). In addition, the transmembrane (TM) domain or glycosylphosphatidylinositol anchor was removed from *PbTRAP* by introducing two stop codons in order to maximize protein secretion from any virus-transduced cell (40).

Prior to immunization animals were anesthetized using an inhalation chamber containing a mixture of gases, isoflurane (23.5%) and oxygen (12 L/min). Mice were primed with simian adenoviral vector 63 (ChAd63) encoding different transgenes at a dose of 10^8^ infectious units (IU) and 8 weeks later boosted with vaccinia virus-modified virus strain Ankara (MVA) encoding the relevant transgene at a concentration of 10^7^ PFU/ml, unless stated otherwise. All viral vector vaccines were administered intramuscularly in endotoxin-free PBS. Vaccinations with two different transgenes in coadministration groups were carried out either with each vaccine injected into the left or right thigh muscle separately or with the mixture of both vaccines administered in a single thigh muscle, as indicated in Results. All recombinant ChAd63 and MVA viral vectors used throughout this study were generated at the Jenner Institute's vector core facility.

### Protein vaccines.

All recombinant protein vaccinations were administered intramuscularly as a 50-μl dose containing sterile PBS and 15 μg of protein formulated with 5 μl of Abisco 100 adjuvant (provided by the Jenner Institute adjuvant bank). For the viral vector and protein mixture group, a 50-μl vaccine dose comprised PBS, MVA expressing the relevant transgene, and 15 μg of the recombinant protein formulated with 5 μl of Abisco 100 adjuvant.

### Whole IgG ELISA.

Enzyme-linked immunosorbent assays (ELISAs) measuring total IgG were carried out as described previously (41). In brief, blood was collected from mice using tail vein bleeds and stored overnight at 4°C. On the following day, the tubes were spun at 13,000 rpm for 4 min, and the serum was collected and stored at 4°C until the assay. Nunc Immuno Maxisorp Plates were coated with protein diluted in PBS to a concentration of 1 μg/ml, followed by incubation overnight at room temperature. Plates were washed with PBS–0.05% Tween 20 (PBS/T) and blocked with 10% skimmed milk powder in PBS/T. Sera were diluted, typically at a starting concentration of 1:100, added into duplicate wells, and serially diluted 3-fold down the plate. Plates were incubated for 2 h at room temperature and then washed as described before. Goat anti-mouse whole IgG conjugated to alkaline phosphatase was added for 1 h at room temperature. After a final wash, *p*-nitrophenylphosphate at 1 mg/ml in diethanolamine buffer was used as a developing substrate. The optical density at 405 nm (OD_405_) was read using a model 550 microplate reader. Serum antibody endpoint titers were taken as the *x* axis intercept of the dilution curve at an absorbance value three standard deviations greater than the OD_405_ for serum from a naive mouse. A standard positive serum sample and naive serum sample were added as controls for each assay. Naive mouse serum was negative for antigen-specific responses against all of the recombinant proteins used.

### Peptides.

Crude 20-mer peptides overlapping by 10 amino acids and representing full-length P. berghei CSP and TRAP were synthesized by Mimotopes. Peptides were reconstituted in dimethyl sulfoxide at a concentration of 50 mg/ml. The peptides were combined into a single pool or three different subpools for their use in intracellular cytokine staining (ICS) and *ex vivo* gamma interferon (IFN-γ) enzyme-linked immunospot (ELISPOT) assays, respectively, at a final individual peptide concentration of 5 μg/ml.

### Splenocyte preparation.

Naive mice were sacrificed by cervical dislocation, and their spleens were removed. The spleens were homogenized in PBS and passed through a 70-μm-pore-size cell strainer, and cells were pelleted by centrifugation at 500 × *g* for 5 min. The cell pellet was then treated with ammonium chloride-potassium (ACK) lysis buffer for 5 min before being washed with 25 ml of PBS. Splenocytes were then immediately centrifuged at 500 × *g* for 5 min, and the resulting pellet was resuspended in 10 ml of complete medium (D10) per spleen. Total number of splenocytes was determined by CASY counter and adjusted to 10^7^ cells per ml of complete medium for use in *ex vivo* IFN-γ ELISPOT assays.

### *Ex vivo* IFN-γ ELISPOT assay.

*Ex vivo* IFN-γ ELISPOT assays were carried out using peripheral blood mononuclear cells (PBMCs) isolated from blood as previously described (42). In brief, nitrocellulose-bottomed 96-well Multiscreen HA filtration plates were coated with anti-mouse IFN-γ monoclonal antibody (MAb) overnight at 4°C. PBMCs were isolated from peripheral blood collected from tail vein bleeds into 200 μl of 10 mM EDTA/PBS. Erythrocytes were lysed using ACK lysis buffer, and PBMCs were harvested using centrifugation. The cells were washed, resuspended in complete medium, and counted using a CASY counter, and 50 μl of culture was plated into duplicate wells. A 50-μl portion of each of the peptide subpools diluted in medium plus 250,000 naive splenocytes was added to test wells as a source of antigen-presenting cells. Medium and naive splenocytes were also plated onto the negative-control wells. Plates were incubated at 37°C in 5% CO_2_ for approximately 18 h. The plates were then washed and incubated with biotinylated anti-mouse-IFN-γ MAb (Mabtech), followed by incubation with a streptavidin alkaline phosphatase polymer. Spots were developed by addition of color development buffer and counted using an ELISPOT reader and accompanying software. Results are expressed as spot-forming units (SFU) per million PBMC. Background responses in medium-only wells were subtracted from those measured in wells stimulated by one of three peptide subpools.

### Isolation of liver-resident lymphocytes.

Livers from mice were perfused with PBS and dissected, mashed, and passed through a 100-μm-pore size filter. The liver cells were centrifuged at 1,350 rpm for 7 min, and the pellet was resuspended in 15 ml of 33% isotonic Percoll and then centrifuged at 693 × *g* for 12 min without brake. Cell debris and the supernatant were removed. The pellet was resuspended in 2 ml of ACK, and the erythrocytes were lysed for 5 min. Then, 25 ml of PBS was added, followed by centrifugation at 1,500 rpm for 5 min. The lymphocytes were washed with complete minimal essential medium (MEM) and resuspended in 500 μl of complete MEM.

### ICS.

For intracellular cytokine staining (ICS), ACK lysis buffer-treated whole-blood PBMCs were incubated for 5.5 to 6 h in the presence of a peptide pool representing the antigen of interest at individual peptide concentrations of 5 μg/ml. Golgi-Plug (BD Biosciences) (1 μl/ml) and anti-CD107a PE (clone eBio/D4B) at a final dilution of 1:200 were also included. Phenotypic analysis of CD8^+^ and CD4^+^ T cells was performed by staining PBMCs using the following antibody clones: anti-CD8 PerCP-Cy5.5 (clone 53-6.7) and eFluor 650-coupled anti-CD4 (GK1.5) and anti-IFN-γ APC (XMG1.2) (BD Biosciences). Also, the surface staining of liver-resident lymphocytes was performed using the following antibodies: anti-NK1.1 FITC, anti-CD3 Alexa Fluor 700, anti-CD69 PE-Cy 7, anti-CD8 eFluor 450 (BD Biosciences). The liver-resident invariant natural killer T cells (iNKT cells) were stained with CD1d tetramer conjugated to PE. Nonspecific binding of antibodies was prevented by incubating with anti-CD16/CD32 Fcγ III/II receptor prior to staining. Flow cytometric analyses were performed using an LSRII instrument. Data were analyzed using either FACSDiva or FlowJo software. Analysis of multifunctional CD8^+^ T-cell responses was performed using Boolean analysis in FlowJo software, Pestle, and SPICE 4.0 kindly provided by M. Roederer (National Institutes of Health, Bethesda, MD).

### *In vivo* CD8^+^ and CD4^+^ T-cell depletions.

The T-cell depletions were performed as described previously (43). Briefly, *in vivo*-depleting MAbs were purified by protein G affinity chromatography columns from hybridoma culture supernatants. Anti-CD4 GK1.5 (rat IgG2a) and anti-CD8 2.43 (rat IgG2a) were sterile filtered and diluted in sterile PBS. Normal rat IgG (nRatIgG) was purchased from Sigma and purified by the same method. For depletion of CD4^+^ or CD8^+^ T cells, mice were injected intraperitoneally with 200 μg of the relevant MAb on days −2 and −1 before and on the day of challenge. Depletion of CD4^+^ and CD8^+^ T cells was confirmed by flow cytometry of surface-stained PBMCs from depleted and control mice a day after the challenge.

### *In vivo* Kupffer and NK cell depletions.

The *in vivo* depletion of liver-resident Kupffer cells was accomplished by intravenous administration of liposome formulated clodronate 48 h prior to challenge at a concentration of 10 μl of suspension per 1 g of mouse (44). Control liposomes containing PBS were also included. To deplete natural killer (NK) cells, mice received a single dose of 200 μl of anti-asialo GM1 antiserum diluted 1:8 in 0.5× PBS on days −2, 0, and +2 relative to challenge, as described previously (45). Naive rabbit serum was also used as a control (kindly provided by A. Douglas). NK cell depletion was confirmed by flow cytometry of surface-stained PBMCs from depleted and control mice 3 days after the challenge.

### Sporozoite neutralization assay.

Sporozoite neutralization assay was performed as described previously (32). At room temperature 50,000 salivary gland wild-type P. berghei sporozoites were incubated with either undigested 3D11 or 3D11 Fab in total of 0.5 ml of RPMI 1640. After a 30-min incubation, 500 μl of ice-cold RPMI 1640 was added, and all sample tubes were placed on ice. Immediately thereafter, 100 μl of culture containing 5,000 sporozoites was injected intravenously (i.v.) into each animal.

### Fragmentation of 3D11.

Fab fragments of 3D11 MAb were generated by incubation with immobilized Ficin in the presence of 25 mM cysteine according to the manufacturer's instructions (Thermo Scientific Pierce). Fab fragments were purified by passing twice over a protein A column. The purity of Fab fragments was verified by nonreducing SDS-PAGE.

### Transgenic (tg) P. berghei expressing firefly luciferase.

The tg P. berghei parasites (PbGFP-Luccon) used in these studies expressed fusion GFP (mutant 3) and firefly luciferase (LucIAV) gene under the control of constitutive EF1a promoter (46). The parasites were generated by a stable double-crossover homologous integration of the transgene into the P230p locus of the P. berghei reference line ANKA clone cl15cy1. The tg parasite was kindly provided by Oliver Billker from Wellcome Trust Sanger Institute, Hinxton, United Kingdom.

### Parasite preparation.

Female A. stephensi mosquitoes were fed on phenylhydrazine (PHZ)-treated female TO mice infected with P. berghei parasites. At 21 days after the feed, the mosquitoes were dissected, the salivary glands were isolated, and the salivary gland sporozoites were extracted and diluted in RPMI 1640. The total number of sporozoites was determined using a hemocytometer, and specified quantities were injected i.v. into mice.

### Thin-blood smears to assess parasitemia.

Thin-blood smears were prepared on glass slides from a drop of blood obtained from a tail clip. The blood smear was allowed to air dry before fixation with methanol and stained for at least 1 h using 5% Giemsa diluted in distilled H_2_O. The slides were then removed and allowed to air dry at room temperature.

### Statistical modeling to predict parasitemia.

To obtain the time taken to reach 1% blood-stage parasitemia, a linear regression model was used, as described previously (29). Briefly, blood parasite counts were obtained for 3 to 5 consecutive days, twice a day, starting on day 4 after challenge. The logarithm to base 10 of the calculated percentage of parasitemia was plotted against time after challenge. The Prism 5 (GraphPad) statistical analysis package was used to generate a linear regression model using the linear part of the blood-stage growth curve. From this, the time taken to reach 1% parasitemia was obtained, expressed in days postchallenge. As expected for vaccines containing only preerythrocytic-stage antigens, all infected mice exhibited similar exponential blood stage growth regardless of treatment group. The time to reach 1% parasitemia was used in survival analyses to assess vaccine efficacy; mice without detectable parasitemia after 15 days were considered to have complete sterile protection. This approach has been used previously; the time to blood-stage parasitemia reflects the number of parasites erupting from the liver, provided there is no blood-stage immunity (47).

### *In vivo* imaging system (IVIS).

The bioluminescent luciferase signal (BLS) was detected by imaging whole animals using the *in vivo* IVIS 200 imaging system, as described previously (48). Briefly, after intravenous injection of tg P. berghei sporozoites, the animals were anesthetized using an isoflurane chamber at a different time points depending on the experiment. Prior to imaging, 100 μl of d-luciferin substrate dissolved in sterile PBS to a concentration of 50 mg/kg was subcutaneously injected into the neck region of mice. Animals were imaged for 60 to 180 s at a binning value of 8 and field of view (FOV) of 12.8 cm, 8 min after the injection of the substrate. Mice remained anesthetized throughout the procedure. Quantification of BLS was performed using Living Image 4.2 image analysis software. Regions of interest were created around the liver area of the mouse body and kept constant for all animals. The measurements were expressed on a log scale as total flux of photons emitted from animals adjusted per second of exposure time.

### Statistics.

GraphPad Prism 5.0 for Mac OS was used for all statistical analysis unless indicated otherwise. The Kolmogorov-Smirnov test for normality was used to determine whether the values followed a Gaussian distribution prior to statistical analysis when comparing two or more populations. An unpaired *t* test was used to compare two normally distributed groups, whereas the Mann-Whitney rank test was used to compare two nonparametric groups. If more than two groups were present, the nonparametric data were compared using the Kruskal-Wallis test with Dunn's multiple-comparison posttest, whereas normally distributed data were analyzed by one-way analysis of variance (ANOVA) with Bonferroni's multiple-comparison posttest. The effect of two variables was explored using two-way ANOVA with Bonferroni's multiple-comparison posttest. Correlation strength was tested using either Pearson's or Spearman's tests as indicated in the results section. Kaplan-Meier survival curves were used to represent protective efficacy to challenge with P. berghei. When required, protection was also assessed using IVIS *in vivo* imaging and data log_10_ transformed prior to analysis. All ELISA titers were also log_10_ transformed before analysis. A *P* value of <0.05 was considered statistically significant (*, *P* < 0.05; **, *P* < 0.01; ***, *P* < 0.001; ****, *P* < 0.0001).

## RESULTS

### Combining virus-vectored CSP and TRAP vaccines fails to improve protective efficacy.

CSP and TRAP vaccines, by themselves, elicit immune responses and some degree of protection (12, 24, 26, 27). In the present study, these vaccines were combined with the aim of providing enhanced immunity in mice to rodent malaria Plasmodium berghei. Virus-vectored vaccines, adenovirus prime and MVA boost (Ad-M) PbCS and Ad-M PbTR, were trialed both separately and together as a combination vaccine on inbred C57BL/6 and outbred CD1 mice. A heterologous prime-boost regimen using replication-deficient ChAd63 (Ad) and modified vaccinia virus strain Ankara (M) with an 8-week interval was used to deliver the antigens. This regime was previously found to optimize immune responses (27, 41).

**FIG 1:**
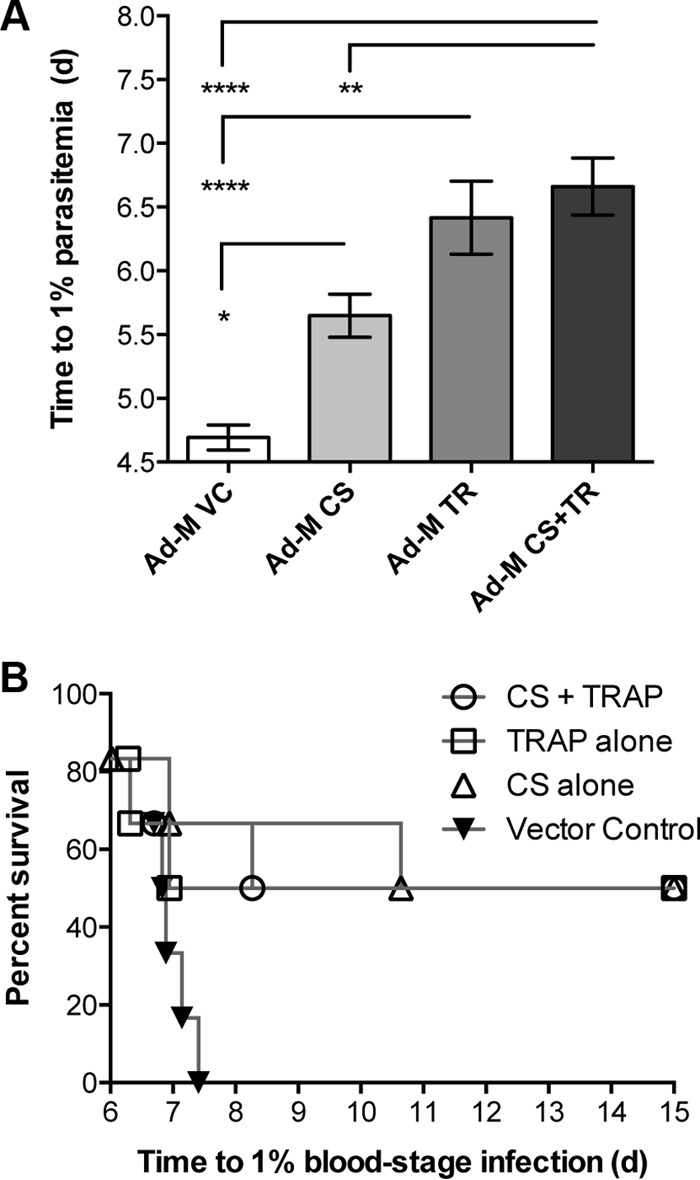
C57BL/6 mice and CD1 outbred mice were vaccinated using a prime-boost strategy. Viral vectors containing either PbTRAP or PbCSP were intramuscularly injected into the thigh muscles of the mice, with 10^8^ IU of ChAd63, followed by MVA at 10^7^ PFU/ml 8 weeks later. Mice vaccinated using the combination vaccine were injected with Ad-M TR in one thigh and Ad-M CSP in the other. The vehicle control used empty ChAd63 and MVA-GFP-OVA. Two weeks after boost vaccination mice were challenged by i.v. injection of 2,000 P. berghei sporozoites. The protective efficacy is here presented as time predicted to reach 1% blood-stage parasitemia using a linear regression model. Blood-stage parasitemia was monitored from day 4 postchallenge. (A) Time to 1% blood-stage parasitemia in C57BL/6 mice (*n* = 6 per group). (B) Time to 1% blood-stage parasitemia in outbred CD1 mice. *, *P* < 0.05; **, *P* < 0.01; ***, *P* < 0.001; ****, *P* < 0.0001 (determined by one-way ANOVA with Bonferroni's multiple-comparison posttest).

The combination vaccine (Ad-M CS + Ad-M TR [Ad-M CS+TR]) produced better protection against sporozoite challenge than either component trialed alone, but this improvement was not significantly better than the protection achieved by Ad-M TR, according to a prediction of time taken to reach 1% blood-stage parasitemia in C57BL/6 after challenge with 2,000 sporozoites (spz) per mouse (Fig. 1A). The Ad-M CS+TR achieved a delay to 1% parasitemia of 6.7 days, while the Ad-M CS and Ad-M TR vaccines yielded delays of 5.6 and 6.4 days, respectively; all three vaccination groups took significantly longer to reach 1% parasitemia than the 4.7 days taken in mice vaccinated with an Ad-M vehicle control (*P* < 0.0001) (Fig. 1A). The levels of sterile protection achieved in CD1 mice showed a similar effect, with 50% of animals vaccinated with Ad-M CS, Ad-M TR, and Ad-M CS+TR vaccines exhibiting sterile protection compared to no sterile protection seen with animals injected with Ad-M vehicle control (Fig. 1B). Since all C57BL/6 showed blood-stage parasitemia, a bar graph was judged the clearest way to present these results, whereas for mixed outcome with CD1, a Kaplan-Meier curve was the most clear.

**FIG 2:**
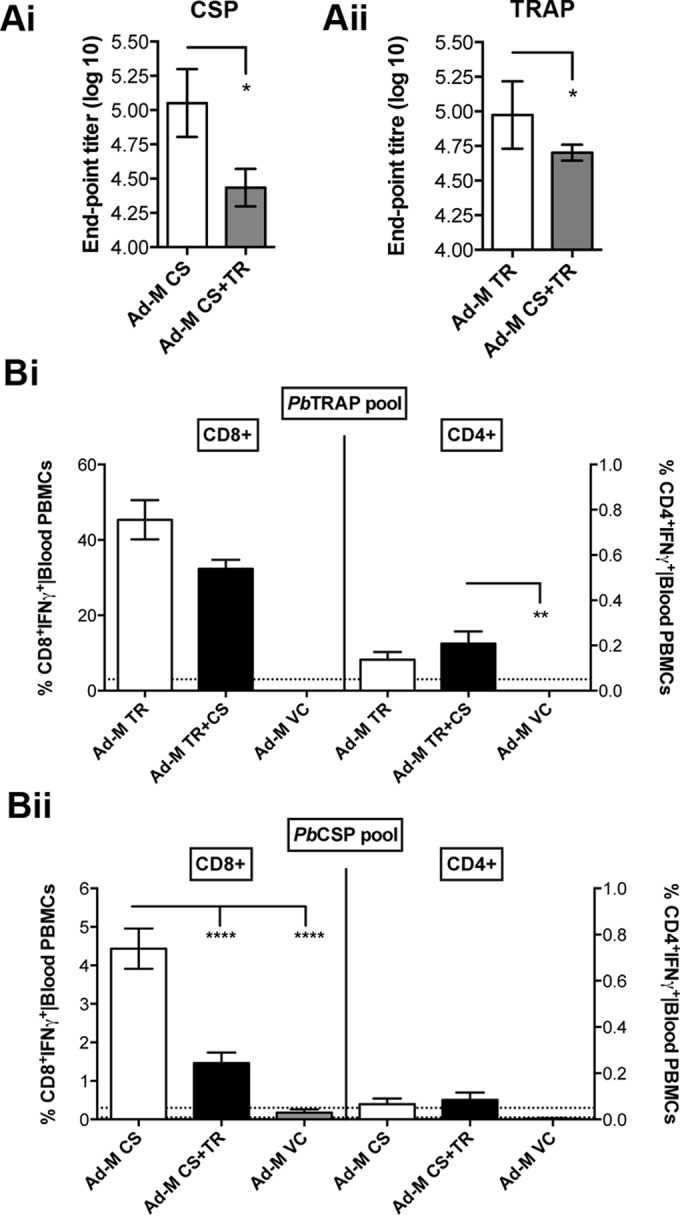
Humoral and cellular responses following Ad-M vaccinations using PbCSP and PbTRAP alone and in combination. C57BL/6 mice were vaccinated as described in Fig. 1. Two weeks after boost administration and 1 day before challenge, the humoral and cellular responses to vaccination were assessed using endpoint ELISA and ICS respectively. (A) Whole IgG Ab titers were measured against PbCSP (i) and PbTRAP (ii) recombinant proteins. No response was seen in this or subsequent ELISAs to serum from vehicle control groups. (B) Whole-blood PBMCs were restimulated with single peptide pools representing the full length of PbTRAP (i) and PbCSP (ii) antigens. The frequencies of CD8^+^ IFN-γ^+^ (left *y* axis) or CD4^+^ IFN-γ^+^ (right *y* axis) T-cell subsets were quantified. *, *P* < 0.05; **, *P* < 0.01; ****, *P* < 0.0001 (as determined by one-way ANOVA with Bonferroni's multiple-comparison posttest).

Humoral and cellular immune responses to the combination vaccine were compared to those of mice vaccinated with the Ad-M CS and Ad-M TR components. Figure 2Ai shows that the combination vaccine elicits significantly lower antibody titers against the CS protein than does the Ad-M CS vaccine used alone; the same is true of anti-TRAP antibody titers when the combination is compared to Ad-M TRAP (Fig. 2Aii). This evidence of antigenic interference could explain why the combination vaccine did not work significantly better than the Ad-M TR vaccine.

Taken together these data suggested that the combination vaccine had some potential but required further optimization. To this end, experiments were performed to elucidate the mechanism of protection of the combination vaccine, as detailed below.

### In the Ad-M CS+TR combination vaccine, protection is mediated by antibodies against PbCS and a CD8^+^ response against PbTRAP.

**FIG 3:**
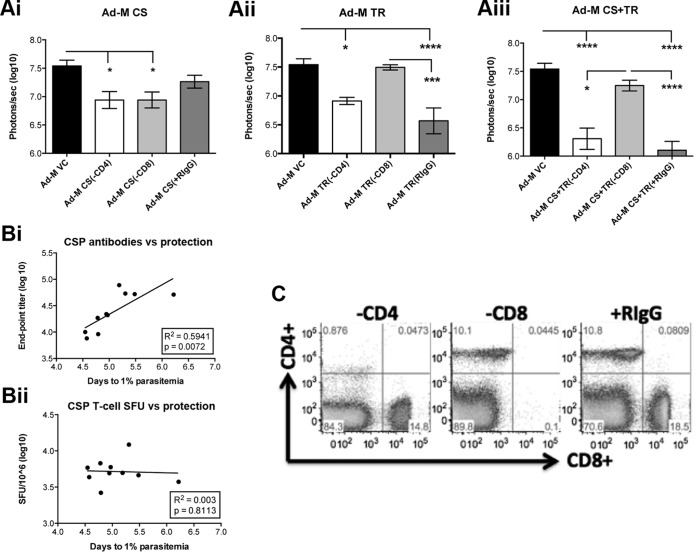
Depletion of CD4^+^ or CD8^+^ T cells in mice vaccinated with Ad-M PbCSP or Ad-M PbTR vaccines. (A) C57BL/6 mice (*n* = 20 per group) were vaccinated with Ad-M CSP (i), Ad-M TR (ii), or a combination of both (iii). A vector control group (Ad-M VC) (*n* = 10) was included. Two weeks after the boost, a selection of animals from each group (*n* = 6 per group) were depleted of CD4^+^ or CD8^+^ T cells, using three 200-μg intraperitoneal injections of rat GK1.5 or 2.43 MAb, respectively, on days −2, −1, and 0 relative to challenge with 5,000 P. berghei GFP/Luc spz. Control rat IgG1 (+RIgG) antibodies were used as an injection control (*n* = 8 per group). At 44 h after challenge, the BLS emitted from infected animals was quantified. Means with the standard errors of the mean (SEM) are shown. *, *P* < 0.05; ***, *P* < 0.001; ****, *P* < 0.0001 (as determined by one-way ANOVA with Bonferroni's multiple-comparison posttest). (B) A strong correlation between time to reach 1% parasitemia (Tto1) and anti-PbCSP Ab titers (i), but not *ex vivo* IFN-γ ELISPOT responses (ii), suggests that protection is mainly mediated by Abs in Ad-M CSP vaccination. (C) Representative FlowJo plots demonstrate the depletion of CD4^+^ or CD8^+^ T cells compared to vaccinated animals that received control rat IgG1.

ICS showed that high CD8^+^ IFN-γ^+^ T-cell responses were seen only in response to PbTRAP, whereas no significant CD4^+^ responses were seen except for a small but detectable response with PbTRAP using the Ad-M CS+TR regime (Fig. 2Bi and Bii). In an experiment examining the mechanisms of protection of the Ad-M CS, Ad-M TR, and Ad-M CS+TR vaccines, either CD8^+^ or CD4^+^ T cells were depleted in vaccinated and control C57BL/6 mice. Neither CD8^+^ nor CD4^+^ T-cell depletion affected liver-stage burden in Ad-M CS-vaccinated mice compared to the control (Fig. 3Ai), and there was a strong correlation between the endpoint CSP Ab titer and the time to 1% blood-stage infection (Fig. 3Bi), suggesting that antibodies help mediate protection in this Ad-M CS regimen. A correlation was not seen between CSP T-cell SFU and time to 1% blood-stage infection (Fig. 3Bii). CD8^+^ but not CD4^+^ T-cell depletion abrogated the protective effect of Ad-M TR (Fig. 3Aii) and Ad-M CS+TR (Fig. 3Aiii) vaccinations, suggesting that CD8^+^ T cells contribute to protection in these regimes; flow cytometry confirmed successful depletion of CD4^+^ and CD8^+^ T cells (Fig. 3C).

### High levels of anti-PbCS Ab are required for protection against P. berghei challenge in C57BL/6 mice.

**FIG 4:**
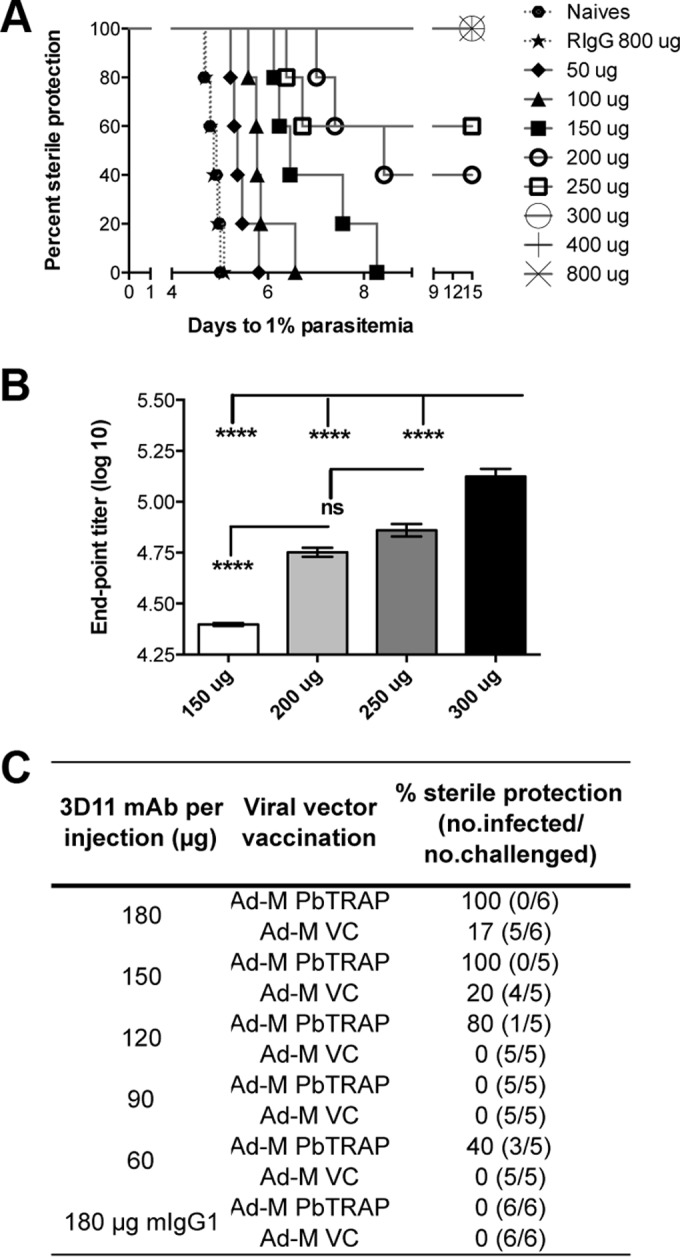
Enhanced protective effect of 3D11 MAb with the Ad-M PbTR vaccine. Naive C57BL/6 mice (*n* = 5 to 6 per group) were injected i.v. with three doses of anti-PbCSP mouse MAb 3D11 on days −3, −2, and −1 relative to the challenge. The mice were then challenged with 2,000 P. berghei spz each. A variety of 3D11 dosage sizes were used; these are labeled on each graph. (A) Linear regression modeling was used to predict Tto1 for parasitized mice. The Tto1 values were used for Kaplan-Meier analysis. Mice that didn't develop blood-stage infection by day 9 still demonstrated sterile protection by day 15 postchallenge. Blood-stage parasitemia was monitored using Giemsa-stained blood films. (B) 3D11 MAb titers from the serum of treated mice were measured by endpoint ELISA, immediately before challenge. (C) C57BL/6 mice (*n* = 5 to 6 per group) were vaccinated using a heterologous prime-boost regime using ChAd63 and MVA viral vectors expressing PbTR. The dosage of 3D11 was varied, in combination with either Ad-M TR or Ad-M VC, and challenged as above. ****, *P* < 0.0001 (as determined by one-way ANOVA with Bonferroni's multiple-comparison posttest). Means with the SEM are shown.

3D11 is an MAb against the repeat region of PbCS (32). It was used to determine the antibody titers required to achieve sterile protection against P. berghei challenge. Administration of 3D11 in three intravenous injections of 300 μg each, producing antibody levels of >5 ELISA units, was sufficient to produce sterile protection against 2,000 sporozoites in all challenged animals (Fig. 4A and B). This demonstrated that high levels of antibody production by a PbCS vaccine would be required to achieve sterile protection of vaccinated mice.

Trialed with Ad-M TR in a combination regime, sterile protection was achieved with much lower dosages of 3D11 than required with 3D11 alone (Fig. 4C). Sterile protection of all mice using the 3D11-TR combination vaccine was achieved with three injections of 150 μg of 3D11 MAb each. This experiment demonstrated that an Ad-M CS+TR vaccine is able to achieve better protection against malaria than the components alone, and thus provided proof of concept for a combination vaccine.

### An Ad-P CS/Ad-M TR combination vaccine confers sterile protection to C57BL/6 mice.

**FIG 5:**
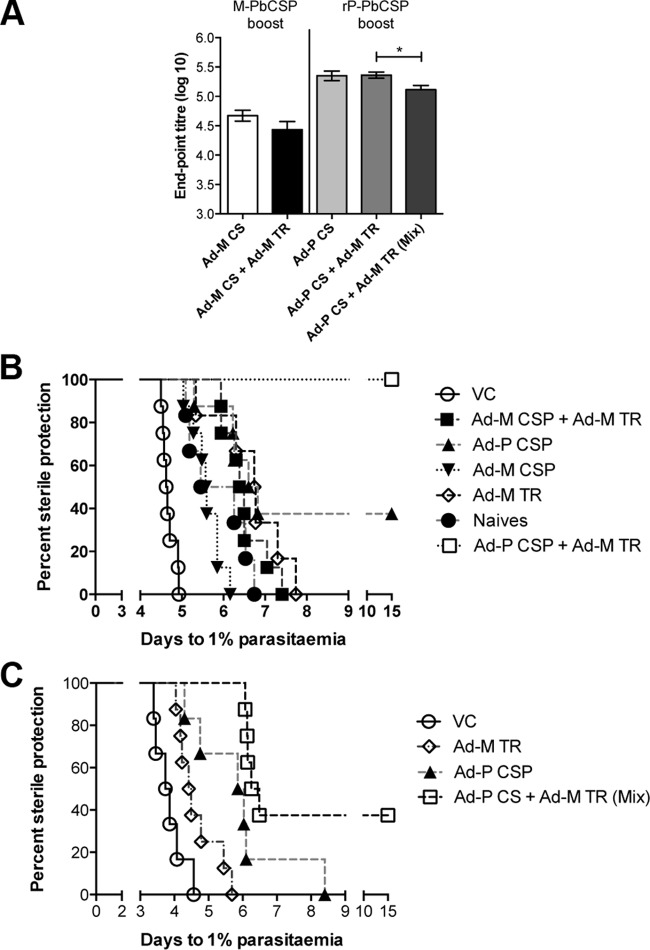
A particulate PbCSP vaccine improves antibody responses and response to challenge in a combination vaccine. C57BL/6 mice (*n* = 6 to 14 per group) were vaccinated with different regimens according to group as follows: naive control group, GFP antigen using ChAd63 prime and MVA boost; Ad-M TR, PbTRAP antigen using ChAd63 prime and MVA boost; Ad-M TR (Ab100), PbTRAP antigen using ChAd63 prime and MVA with Abisco 100 boost; Ad-P CSP, PbCSP antigen using ChAd63 prime and 15 μg PbCSP in Abisco 100 boost; and Ad-M PbCSP, PbCSP antigen using ChAd63 prime and MVA boost. In the “Ad-P CS+Ad-M TR(Mix)” combination, the vaccines were mixed before being injected into left and right legs. In the “Ad-M CS+Ad-M TR” combination vaccine, the PbCSP vaccines were injected into the left leg, and the PbTRAP vaccines were injected into the right leg. (A) PbCSP-specific whole IgG serum Ab titers measured 2 weeks after boost by endpoint ELISA. *, *P* < 0.05 (as determined by unpaired *t* test). Means with the SEM are shown. Two weeks after boost mice were challenged with 2,000 P. berghei. (B and C) Kaplan-Meier survival curves based on predicted time to 1% parasitemia.

Using an Ad-P CS vaccine with 15 μg of recombinant PbCS in Abisco 100 as a boost produced almost 10-fold-higher titers of anti-PbCS antibody than did the Ad-M CS vaccine (Fig. 5A). This order-of-magnitude increase was maintained when the Ad-P CSP vaccine was combined with the Ad-M TR vaccine. Unlike with the Ad-M CSP+TR combination vaccine, no antigenic interference was seen when Ad-P CSP and Ad-M TR were injected into separate sites. A slight but statistically significant decrease in antibody titers was seen when Ad-P CS and Ad-M TR were mixed and injected into the same site (Fig. 5A).

The Ad-P CS vaccine, despite achieving anti-PbCS Ab titers as high as conferred sterile protection using 3D11 MAb, conferred sterile protection on a nonsignificant number of mice in one experiment (Fig. 5B) and on no mice in another (Fig. 5C) after challenge with 2,000 P. berghei spz.

The combination regimen, whether CSP and TRAP vaccines were injected into separate legs (Fig. 5B) or mixed and coinjected (Fig. 5C), consistently conferred superior levels of sterile protection compared to the Ad-P CSP or Ad-M TR vaccines alone. Ad-P CS and Ad-M TR administered as a combination vaccine into separate sites produced sterile protection in 100% of mice, suggesting that the increase in antibody titers caused by Ad-P CS compared to Ad-M CS was sufficient to improve the combination CS-TR vaccine to the desired level of efficacy, as suggested by the results given above (Fig. 4C). Thus, the CS+TR vaccine was successfully tailored to achieve the desired results.

### In the 3D11+Ad-M TR combination vaccine, protection is mediated by antibodies in an Fc-independent fashion. NK cells and CD8^+^ T cells are important mediators of protection.

The mechanisms responsible for the effectiveness of the Ad-P CS+Ad-M TR combination vaccine were examined. These mechanisms were investigated using 3D11 MAb instead of Ad-P CS since it was not practical to optimize the Ad-P CS dosage to a level required to maximize sensitivity in these experiments.

**FIG 6:**
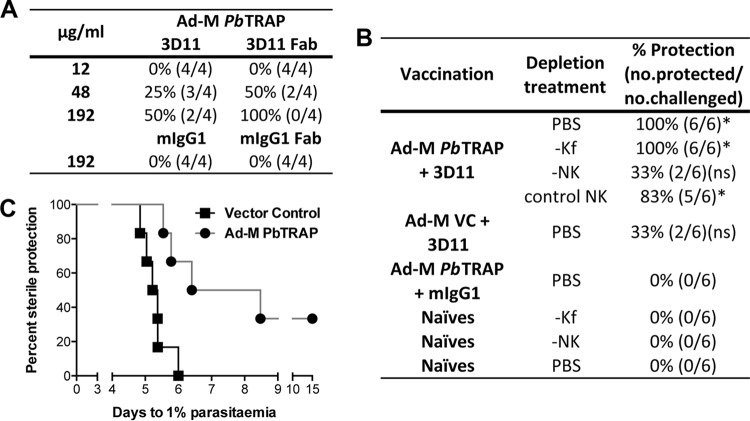
Immunological mechanisms of the 3D11 and Ad-M PbTRAP combination vaccine. C57BL/6 mice (*n* = 6 per group) were vaccinated with a heterologous prime-boost regimen using ChAd63 and MVA viral vectors expressing PbTRAP. A control group (*n* = 6) was vaccinated with empty vector control viruses. (A) The role of the Fc region in protection was investigated using a sporozoite neutralization assay. A total of 50,000 P. berghei spz were incubated with either intact or Fab fragments of 3D11 at a range of concentrations. A total of 5,000 sporozoites, each opsonized with either 3D11 or 3D11 Fab, were then i.v. injected into Ad-M PbTRAP-vaccinated animals. The percentages of animals experiencing sterile protection are shown. (B) Two weeks after boost and on days −3, −2, and −1 relative to challenge with 2,000 P. berghei spz, vaccinated animals were injected with 120 μg of 3D11 MAb each day. Control mIgG1 was administered in an identical manner. Additionally, either NK (−NK) or Kupffer (−Kf) cells in the vaccinated animals were depleted by multiple i.v. injections of either anti-asialo GM1 or clodronate liposomes, respectively, prior to challenge. An NK depletion control was included. The levels of sterile protection were used to quantify the effects of different treatments on vaccine-induced protection. *, *P* < 0.05; ns, nonsignificant [as determined by Fisher exact test comparing the indicated group to the (Ad-M PbTRAP)+mIgG1 group]. (C) C57BL/6 mice were immunized with Ad-M PbTRAP or empty viral Ad-M VC vaccines (*n* = 6 per group). Two weeks after boost animals were challenged with 200 P. berghei spz. Kaplan-Meier survival curves were determined based on the predicted time to 1% parasitemia.

Removal of the Fc portion of the 3D11 MAb had no effect on levels of sterile protection compared to intact 3D11 in a sporozoite neutralization assay (Fig. 6A). This suggests that the mechanism of protection of 3D11 in this combination vaccine is not via Fc-dependent phagocytosis by Kupffer cells or other macrophages. It could, however, still be mediated by Fc-independent phagocytosis.

Depleting Kupffer cells had no significant effect on the protection conferred by the Ad-M TR+3D11 vaccine (Fig. 6B). Depleting NK cells, on the other hand, did decrease the protection associated with this vaccine (Fig. 6B), suggesting that NK cells may play an important role in the mechanism of protection.

**FIG 7:**
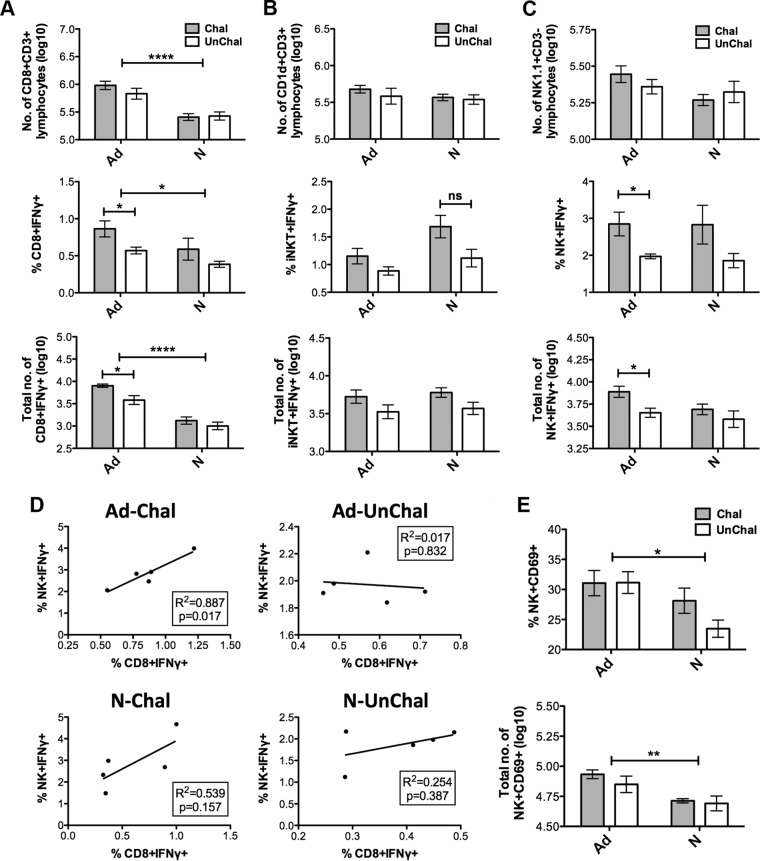
ICS analysis of liver-resident lymphocytes. C57BL/6 mice (*n* = 5 per group) were immunized with a single ChAd63-PbTRAP vaccine (Ad) and 2 weeks later challenged with 3,000 P. berghei spz. At 24 h after challenge, the livers from vaccinated challenged (Ad-Chal) and unchallenged (Ad-UnChal) animals were extracted, and lymphocytes were purified and quantified. Livers from naive challenged (N-Chal) and unchallenged (N-UnChal) animals were also included. ICS was performed, and samples were acquired using a BD LSRII flow cytometry instrument. The total number per mouse liver of CD8^+^ T cells (A), iNKT cells (B), and NK cells (C) was quantified (top panel), as well as the percentage (middle panel) and total number (bottom panel) of IFN-γ positive cells within each subset for all treatment groups. (D) A strong correlation was obtained between the proportions of CD8^+^ IFN-γ^+^ and NK^+^ IFN-γ^+^ cells for the Ad-UnChal group, but not other groups. (E) The percentage (top panel) and total number (bottom panel) of NK^+^ CD69^+^ cells per liver for each treatment group were determined. ****, *P* < 0.0001; **, *P* < 0.01; *, *P* < 0.05; ns, nonsignificant (as determined by two-way ANOVA). Means with the SEM are shown.

ICS analysis of three cell types from the livers of vaccinated and unvaccinated and then challenged and unchallenged mice provided further data on the mechanisms of protection. Vaccinated mice were found to have more CD8^+^ CD3^+^ T cells than naive mice and that, of these vaccinated mice, challenged mice have a higher proportion and higher absolute numbers of CD8^+^ IFN-γ^+^ cells (Fig. 7A). No significant difference in the numbers or proportions of CD1d^+^ CD3^+^ iNKT cells were found between vaccinated or challenged mice (Fig. 7B). However, NK cells which are IFN-γ^+^ were found to be present in higher numbers and in a higher proportion in vaccinated and challenged mice than in vaccinated and unchallenged mice (Fig. 7C). There was found to be a correlation between NK^+^ IFN-γ^+^ cell percentage and CD8^+^ IFN-γ^+^ cells in vaccinated and challenged mice only and not in other groups (Fig. 7D). From this and the fact that there was found to be a higher proportion and higher absolute numbers of CD69^+^ NK cells in vaccinated compared to nonvaccinated mice (Fig. 7E), it appeared that NK cells could be important in the mechanism of protection conferred by Ad-M TR.

These data, together with other data presented above (Fig. 3), suggest a model for the mechanism of protection of this combination vaccine whereby 3D11+Ad-M TR confers sterile protection by PbCSP-specific antibodies reducing the number of sporozoites reaching the liver and CD8^+^ T cells destroying any hepatocytes infected by the few remaining sporozoites. With the Ad-P CSP+Ad-M TR regime, protection may also be mediated by CSP-specific CD8^+^ T cells and IFN-γ, as found in other studies (33, 49–51); however, this was not examined here. A prediction of this model is that Ad-M TR alone should confer sterile protection when fewer sporozoites are used in a challenge. This was found to be the case: in a challenge with 200 sporozoites, Ad-M TR was able to confer sterile protection to 38% of challenged mice (Fig. 6C).

## DISCUSSION

Currently, the most protective preerythrocytic-stage malaria vaccine candidates in human clinical trials—RTS,S and Ad-M ME.TRAP—rely on the induction of high Ab titers and powerful T-cell responses, against CS and TRAP proteins, respectively. However, individually both vaccines elicit only suboptimal protection against experimental malaria sporozoite challenge. Here, a P. berghei mouse malaria model was used to assess the efficacy of a vaccine combining both antigens.

Initially a heterologous chimpanzee adenovirus 63 (Ad) and modified vaccinia virus strain Ankara (MVA) prime-boost strategy (Ad-M) was used with both PbCS and PbTR antigens. The Ad-M regimen has been demonstrated to induce strong humoral and cellular immune responses in mice (29, 43), rabbits (40), rhesus macaques (52), and humans, showing an excellent safety profile in the latter (53, 54) using different malarial antigens. The combination of these antigens failed to induce significantly superior protection compared to the most protective single antigen immunization, however.

The degree of protection observed with Ad-M CS+TR combination vaccine in C57BL/6 mice was substantially lower than that seen in a study by Schneider et al. (55), where DNA/MVA prime-boost vaccination employing the combination of the same PbCS and PbTRAP antigens elicited 60 and 100% sterile protection against 1,000 and 200 P. berghei sporozoite challenges, respectively. However, in the latter case at the time of boosting MVA was administered intravenously, which is a more protective mode of vaccination. Since the intravenous injection of MVA viral vectors is not permitted in the clinical setting, the intramuscular route of administration was chosen.

A previous study using human adenovirus serotype 5 combined with WR vaccinia virus encoding P. yoelii CSP (25) detected higher levels of protection than found here using an Ad-M CSP regime with P. berghei, probably because of the greater immunogenicity of WR vaccinia virus. In a direct comparison, chimpanzee adenoviruses have been shown to be as immunogenic or more immunogenic than human adenovirus vaccine vectors (56, 57).

Since a trend toward increased immunity was observed when PbTRAP and PbCS antigens were coadministered into separate sites, it seemed likely that this particular combination could offer a high degree of protective efficacy if it was carefully tailored for the concurrent induction of higher Ab and T-cell responses. The MAb 3D11 specific against the central repeat region of PbCS antigen was used to determine anti-PbCS Ab requirements necessary for sterile protection in a C57BL/6 animal model. As expected, a clear dose-dependent effect was observed, and an extremely high concentration of anti-CS 3D11 was required for complete sterile protection. Substantially smaller amounts of 3D11 were sufficient for sterile protection if the MAb vaccinations were performed on previously Ad-M PbTRAP-immunized animals.

Thus, the heterologous Ad prime/protein-in-adjuvant boost (Ad-P) regimen for the administration of PbCS antigen was explored, since reports have previously suggested that Ad-P regimen induces significantly higher Ab responses compared to these regimens administered individually (52, 58, 59). Immunization of C57BL/6 mice using the Ad-P PbCSP and Ad-M PbTRAP combination vaccine conferred an exceptionally high level of sterile protection when each antigen was administered into separate sites.

Interestingly, an Ad-P PbCSP and Ad-M PbTRAP combination vaccine administered as a mixture into a single site at the time of prime and boost was less protective than a separate-site vaccination strategy. The mixing of vaccines prior to single-site administration seemed to increase antigenic interference between PbTRAP and PbCSP immunogens, resulting in diminished immune responses against individual antigens, which, in part, could explain the lower level of protective efficacy (60). However, the challenge against the mixed and coinjected vaccination regime was more rigorous, as evident from the lower time taken to reach 1% blood-stage parasitemia in naive mice; this, too, could explain the lower level of protective efficacy. In either case, the combination of CSP and TRAP vaccines substantially enhanced protection compared to individual immunizations. Trials combining P. falciparum CSP and TRAP with other antigens (61, 62) have shown limited protection to date, though these trials did not use the most protective Ad-M or Ad-P regimes.

The cell depletion experiments performed on Ad-M PbTRAP- and 3D11 MAb-immunized mice demonstrated that the protection provided by this combination vaccine was mediated by anti-PbCS Abs capable of neutralizing a large proportion of spz, with PbTRAP-specific CD8^+^ T cells and NK cells providing an important second line of defense against parasites that manage to establish intrahepatic infection. The protection provided by highly protective irradiated spz vaccination has also been shown to be dependent on cellular and humoral immune responses against various spz-stage antigens (63). However, the results obtained using anti-asialo GM1 depleting polyclonal Abs should be interpreted cautiously, since the specificity of these Abs has been questioned (64, 65), and these antibodies may also reduce CD8^+^ T-cell numbers, leading to diminished efficacy.

Anti-CSP Ab-mediated sporozoite neutralization could be accomplished by various mechanisms: complement-dependent lysis, receptor-mediated phagocytosis, and/or perhaps inhibition of sporozoite binding to the liver (66). Touray et al. demonstrated an almost complete lysis *in vitro* of P. gallinaceum sporozoites by anti-sporozoite Abs and mammalian serum compared to only 11 to 15% lysis observed using nonimmune serum (67). The serum samples from RTS,S-immunized volunteers contained opsonizing abs and these antibodies significantly enhanced *in vitro* endocytic activity of the FcR(+) THP-1 human monocyte line in protected volunteers compared to those susceptible to sporozoite infection (68). Complement fixation and endocytosis of opsonized sporozoites relies in part on the Fc region of the Ab. However, in the present study no such role for the Fc region was discovered. In fact, the Fc-independent neutralization of P. berghei sporozoites is in agreement with a previous observation where comparable levels of neutralization were achieved upon opsonization of P. berghei sporozoites with either intact or Fab fragments of 3D11 (32). However, it remains unclear whether Fc-independent phagocytosis was occurring and, if so, what was its contribution to the protection induced by the Ad-M TR+3D11 combination vaccine.

The decreased level of protection obtained after depletion of NK cells is consistent with studies performed by Doolan and Hoffman (69), where protective immunity by irradiated P. yoelii sporozoites or plasmid DNA-vaccinated mice was mediated by effector CD8^+^ T cells and NK cells and depended on interleukin-12 (IL-12), IFN-γ, and nitric oxide (NO). Moreover, in a follow-up study, the same authors established that perforin, granzyme B, and Fas L (Fas ligand) were not required for the protection induced by irradiated P. yoelii sporozoites (45). These authors suggested that the protective response is initiated by antigen-specific CD8^+^ T cells that upon recognition of specific peptide-MHC complexes secrete IFN-γ, which, in turn, induces IL-12 secretion from innate immune cells, such as macrophages, dendritic cells, or monocytes. The IL-12 subsequently stimulates the NK cells, which then may directly kill infected hepatocytes or induce antiparasitic activity through the secretion of IFN-γ. The fact that the depletion of NK cells significantly abrogated protection in this study provides some support for this model. This could also explain the strong correlation between CD8^+^ IFN-γ^+^ and NK^+^ IFN-γ^+^ lymphocyte subsets that was observed and is consistent with publications suggesting that NK-specific IFN-γ responses may be tightly associated with the T-cell responses at the preerythrocytic stage (70), as well as the blood stage (71), of infection. The cytotoxic activity of CD8^+^ T cells has also been demonstrated to be one of the principal mediators of protection against sporozoite challenge (45, 72).

Significantly higher numbers of CD8^+^ T cells were measured in the livers of mice that were immunized with ChAd63-PbTRAP than in the livers of naive animals. Potent TRAP-specific cellular immune responses were also measured 2 weeks after the ChAd63-PbTRAP prime by *ex vivo* IFN-γ ELISPOT assay.

Previous studies have found a role for cytotoxic CD8^+^ T cells and IFN-γ in mediating protection, including with CSP (33, 49–51). These could play a role in the protection mediated by the vaccine regimens presented here: although depletion of CD4^+^ and CD8^+^ T cells did not abrogate the protective effect of the Ad-M CSP vaccine, this may be because anti-CSP antibodies played the principal role in protection, as evident from the strong correlation with time to reach 1% parasitemia (Fig. 3Bi).

In summary, a highly protective vaccination regime was developed by combining two suboptimal subunit vaccines, CSP and TRAP, in a P. berghei malaria mouse model. The successful vaccination regime combined Ad-P CS and Ad-M TR. This elicited strong Ab responses against CS and CD8^+^ T-cell responses against TRAP, reducing the number of sporozoites reaching the liver and causing the destruction of the lower number of infected hepatocytes.

## References

[1] World Health Organization. 2014 World malaria report 2013. World Health Organization. Geneva, Switzerland.

[2] Penny MA, Maire N, Studer A, Schapira A, Smith TA 2008 What should vaccine developers ask? Simulation of the effectiveness of malaria vaccines. PLoS One 3:e3193. 18784833 10.1371/journal.pone.0003193 PMC2527129

[3] Maire N, Tediosi F, Ross A, Smith T 2006 Predictions of the epidemiologic impact of introducing a pre-erythrocytic vaccine into the expanded program on immunization in sub-Saharan Africa. Am J Trop Med Hyg 75:111–118. 16931822 10.4269/ajtmh.2006.75.111

[4] Clyde DF 1975 Immunization of man against falciparum and vivax malaria by use of attenuated sporozoites. Am J Trop Med Hyg 24:397–401. 808142 10.4269/ajtmh.1975.24.397

[5] Hoffman SL, Goh LML, Luke TC, Schneider I, Le TP, Doolan DL, Sacci J, la Vega de P, Dowler M, Paul C, Gordon DM, Stoute JA, Church LWP, Sedegah M, Heppner DG, Ballou WR, Richie TL 2002 Protection of humans against malaria by immunization with radiation-attenuated Plasmodium falciparum sporozoites. J Infect Dis 185:1155–1164. doi:10.1086/339409. 11930326

[6] Seder RA, Chang L-J, Enama ME, Zephir KL, Sarwar UN, Gordon IJ, Holman LA, James ER, Billingsley PF, Gunasekera A, Richman A, Chakravarty S, Manoj A, Velmurugan S, Li M, Ruben AJ, Li T, Eappen AG, Stafford RE, Plummer SH, Hendel CS, Novik L, Costner PJM, Mendoza FH, Saunders JG, Nason MC, Richardson JH, Murphy J, Davidson SA, Richie TL, Sedegah M, Sutamihardja A, Fahle GA, Lyke KE, Laurens MB, Roederer M, Tewari K, Epstein JE, Sim BKL, Ledgerwood JE, Graham BS, Hoffman SL, The VRC 312 Study Team. 2013 Protection against malaria by intravenous immunization with a nonreplicating sporozoite vaccine. Science 341:1359–1365. doi:10.1126/science.1241800. 23929949

[7] Mueller A-K, Labaied M, Kappe SHI, Matuschewski K 2005 Genetically modified Plasmodium parasites as a protective experimental malaria vaccine. Nature 433:164–167. doi:10.1038/nature03188. 15580261

[8] van Dijk MR, Douradinha B, Franke-Fayard B, Heussler V, van Dooren MW, van Schaijk B, van Gemert G-J, Sauerwein RW, Mota MM, Waters AP, Janse CJ 2005 Genetically attenuated, P36p-deficient malarial sporozoites induce protective immunity and apoptosis of infected liver cells. Proc Natl Acad Sci U S A 102:12194–12199. doi:10.1073/pnas.0500925102. 16103357 PMC1189305

[9] Aly ASI, Mikolajczak SA, Rivera HS, Camargo N, Jacobs-Lorena V, Labaied M, Coppens I, Kappe SHI 2008 Targeted deletion of SAP1 abolishes the expression of infectivity factors necessary for successful malaria parasite liver infection. Mol Microbiol 69:152–163. doi:10.1111/j.1365-2958.2008.06271.x. 18466298 PMC2615191

[10] Vaughan AM, O'Neill MT, Tarun AS, Camargo N, Phuong TM, Aly ASI, Cowman AF, Kappe SHI 2009 Type II fatty acid synthesis is essential only for malaria parasite late liver stage development. Cell Microbiol 11:506–520. doi:10.1111/j.1462-5822.2008.01270.x. 19068099 PMC2688669

[11] Butler NS, Schmidt NW, Vaughan AM, Aly AS, Kappe SHI, Harty JT 2011 Superior antimalarial immunity after vaccination with late liver stage-arresting genetically attenuated parasites. Cell Host Microbe 9:451–462. doi:10.1016/j.chom.2011.05.008. 21669394 PMC3117254

[12] Agnandji ST, Lell B, Soulanoudjingar SS, Fernandes JF, Abossolo BP, Conzelmann C, Methogo BGNO, Doucka Y, Flamen A, Mordmüller B, Issifou S, Kremsner PG, Sacarlal J, Aide P, Lanaspa M, Aponte JJ, Nhamuave A, Quelhas D, Bassat Q, Mandjate S, Macete E, Alonso P, Abdulla S, Salim N, Juma O, Shomari M, Shubis K, Machera F, Hamad AS, Minja R, Mtoro A, Sykes A, Ahmed S, Urassa AM, Ali AM, Mwangoka G, Tanner M, Tinto H, D'Alessandro U, Sorgho H, Valea I, Tahita MC, Kaboré W, Ouédraogo S, Sandrine Y, Guiguemdé RT, Ouédraogo JB, Hamel MJ, Kariuki S, Odero C, Oneko M, Otieno K, Awino N, Omoto J, Williamson J, Muturi-Kioi V, Laserson KF, Slutsker L, Otieno W, Otieno L, Nekoye O, Gondi S, Otieno A, Ogutu B, Wasuna R, Owira V, Jones D, Onyango AA, Njuguna P, Chilengi R, Akoo P, Kerubo C, Gitaka J, Maingi C, Lang T, Olotu A, Tsofa B, Bejon P, Peshu N, Marsh K, Owusu-Agyei S, Asante KP, Osei-Kwakye K, Boahen O, Ayamba S, Kayan K, Owusu-Ofori R, Dosoo D, Asante I, Adjei G, Chandramohan D, Greenwood B, Lusingu J, Gesase S, Malabeja A, Abdul O, Kilavo H, Mahende C, Liheluka E, Lemnge M, Theander T, Drakeley C, Ansong D, Agbenyega T, Adjei S, Boateng HO, Rettig T, Bawa J, Sylverken J, Sambian D, Agyekum A, Owusu L, Martinson F, Hoffman I, Mvalo T, Kamthunzi P, Nkomo R, Msika A, Jumbe A, Chome N, Nyakuipa D, Chintedza J, Ballou WR, Bruls M, Cohen J, Guerra Y, Jongert E, Lapierre D, Leach A, Lievens M, Ofori-Anyinam O, Vekemans J, Carter T, Leboulleux D, Loucq C, Radford A, Savarese B, Schellenberg D, Sillman M, Vansadia P, Clinical Trials Partnership RTSS 2011 First results of phase 3 trial of RTS,S/AS01 malaria vaccine in African children. N Engl J Med 365:1863–1875. doi:10.1056/NEJMoa1102287. 22007715

[13] Dunachie SJ, Walther M, Epstein JE, Keating S, Berthoud T, Andrews L, Andersen RF, Bejon P, Goonetilleke N, Poulton I, Webster DP, Butcher G, Watkins K, Sinden RE, Levine GL, Richie TL, Schneider J, Kaslow D, Gilbert SC, Carucci DJ, Hill AVS 2006 A DNA prime-modified vaccinia virus Ankara boost vaccine encoding thrombospondin-related adhesion protein but not circumsporozoite protein partially protects healthy malaria-naive adults against Plasmodium falciparum sporozoite challenge. Infect Immun 74:5933–5942. doi:10.1128/IAI.00590-06. 16988273 PMC1594937

[14] Holder AA 2009 The carboxy-terminus of merozoite surface protein 1: structure, specific antibodies and immunity to malaria. Parasitology 136:1445–1456. doi:10.1017/S0031182009990515. 19627632

[15] Ogutu BR, Apollo OJ, McKinney D, Okoth W, Siangla J, Dubovsky F, Tucker K, Waitumbi JN, Diggs C, Wittes J, Malkin E, Leach A, Soisson LA, Milman JB, Otieno L, Holland CA, Polhemus M, Remich SA, Ockenhouse CF, Cohen J, Ballou WR, Martin SK, Angov E, Stewart VA, Lyon JA, Heppner DG, Withers MR, MSP-1 Malaria Vaccine Working Group. 2009 Blood stage malaria vaccine eliciting high antigen-specific antibody concentrations confers no protection to young children in Western Kenya. PLoS One 4:e4708. doi:10.1371/journal.pone.0004708. 19262754 PMC2650803

[16] Dutta S, Sullivan JS, Grady KK, Haynes JD, Komisar J, Batchelor AH, Soisson L, Diggs CL, Heppner DG, Lanar DE, Collins WE, Barnwell JW 2009 High antibody titer against apical membrane antigen-1 is required to protect against malaria in the *Aotus* model. PLoS One 4:e8138. doi:10.1371/journal.pone.0008138. 19997632 PMC2780715

[17] Douglas AD, Williams AR, Illingworth JJ, Kamuyu G, Biswas S, Goodman AL, Wyllie DH, Crosnier C, Miura K, Wright GJ, Long CA, Osier FH, Marsh K, Turner AV, Hill AVS, Draper SJ 2011 The blood-stage malaria antigen PfRH5 is susceptible to vaccine-inducible cross-strain neutralizing antibody. Nat Commun 2:601. doi:10.1038/ncomms1615. 22186897 PMC3504505

[18] Singh AP, Puri SK, Chitnis CE 2002 Antibodies raised against receptor-binding domain of Plasmodium knowlesi Duffy binding protein inhibit erythrocyte invasion. Mol Biochem Parasitol 121:21–31. doi:10.1016/S0166-6851(02)00017-8. 11985860

[19] Grimberg BT, Udomsangpetch R, Xainli J, McHenry A, Panichakul T, Sattabongkot J, Cui L, Bockarie M, Chitnis C, Adams J, Zimmerman PA, King CL 2007 Plasmodium vivax invasion of human erythrocytes inhibited by antibodies directed against the Duffy binding protein. PLoS Med 4:e337. doi:10.1371/journal.pmed.0040337. 18092885 PMC2140086

[20] Wu Y, Ellis RD, Shaffer D, Fontes E, Malkin EM, Mahanty S, Fay MP, Narum D, Rausch K, Miles AP, Aebig J, Orcutt A, Muratova O, Song G, Lambert L, Zhu D, Miura K, Long C, Saul A, Miller LH, Durbin AP 2008 Phase 1 trial of malaria transmission blocking vaccine candidates Pfs25 and Pvs25 formulated with montanide ISA 51. PLoS One 3:e2636. doi:10.1371/journal.pone.0002636. 18612426 PMC2440546

[21] Williamson KC, Keister DB, Muratova O, Kaslow DC 1995 Recombinant Pfs230, a Plasmodium falciparum gametocyte protein, induces antisera that reduce the infectivity of Plasmodium falciparum to mosquitoes. Mol Biochem Parasitol 75:33–42. doi:10.1016/0166-6851(95)02507-3. 8720173

[22] Farrance CE, Rhee A, Jones RM, Musiychuk K, Shamloul M, Sharma S, Mett V, Chichester JA, Streatfield SJ, Roeffen W, van de Vegte-Bolmer M, Sauerwein RW, Tsuboi T, Muratova OV, Wu Y, Yusibov V 2011 A plant-produced Pfs230 vaccine candidate blocks transmission of Plasmodium falciparum. Clin Vaccine Immunol 18:1351–1357. doi:10.1128/CVI.05105-11. 21715576 PMC3147341

[23] Chowdhury DR, Angov E, Kariuki T, Kumar N 2009 A potent malaria transmission blocking vaccine based on codon harmonized full-length Pfs48/45 expressed in Escherichia coli. PLoS One 4:e6352. doi:10.1371/journal.pone.0006352. 19623257 PMC2709910

[24] Agnandji ST, Lell B, Fernandes JF, Abossolo BP, Methogo BGNO, Kabwende AL, Adegnika AA, Mordmüller B, Issifou S, Kremsner PG, Sacarlal J, Aide P, Lanaspa M, Aponte JJ, Machevo S, Acacio S, Bulo H, Sigauque B, Macete E, Alonso P, Abdulla S, Salim N, Minja R, Mpina M, Ahmed S, Ali AM, Mtoro AT, Hamad AS, Mutani P, Tanner M, Tinto H, D'Alessandro U, Sorgho H, Valea I, Bihoun B, Guiraud I, Kaboré B, Sombié O, Guiguemdé RT, Ouédraogo JB, Hamel MJ, Kariuki S, Oneko M, Odero C, Otieno K, Awino N, McMorrow M, Muturi-Kioi V, Laserson KF, Slutsker L, Otieno W, Otieno L, Otsyula N, Gondi S, Otieno A, Owira V, Oguk E, Odongo G, Woods JB, Ogutu B, Njuguna P, Chilengi R, Akoo P, Kerubo C, Maingi C, Lang T, Olotu A, Bejon P, Marsh K, Mwambingu G, Owusu-Agyei S, Asante KP, Osei-Kwakye K, Boahen O, Dosoo D, Asante I, Adjei G, Kwara E, Chandramohan D, Greenwood B, Lusingu J, Gesase S, Malabeja A, Abdul O, Mahende C, Liheluka E, Malle L, Lemnge M, Theander TG, Drakeley C, Ansong D, Agbenyega T, Adjei S, Boateng HO, Rettig T, Bawa J, Sylverken J, Sambian D, Sarfo A, Agyekum A, Martinson F, Hoffman I, Mvalo T, Kamthunzi P, Nkomo R, Tembo T, Tegha G, Tsidya M, Kilembe J, Chawinga C, Ballou WR, Cohen J, Guerra Y, Jongert E, Lapierre D, Leach A, Lievens M, Ofori-Anyinam O, Olivier A, Vekemans J, Carter T, Kaslow D, Leboulleux D, Loucq C, Radford A, Savarese B, Schellenberg D, Sillman M, Vansadia P, RTSS Clinical Trials Partnership. 2012 A phase 3 trial of RTS,S/AS01 malaria vaccine in African infants. N Engl J Med 367:2284–2295. doi:10.1056/NEJMoa1208394. 23136909 PMC10915853

[25] Bruña-Romero O, González-Aseguinolaza G, Hafalla JC, Tsuji M, Nussenzweig RS 2001 Complete, long-lasting protection against malaria of mice primed and boosted with two distinct viral vectors expressing the same plasmodial antigen. Proc Natl Acad Sci U S A 98:11491–11496. doi:10.1073/pnas.191380898. 11553779 PMC58757

[26] O'Hara GA, Duncan CJA, Ewer KJ, Collins KA, Elias SC, Halstead FD, Goodman AL, Edwards NJ, Reyes-Sandoval A, Bird P, Rowland R, Sheehy SH, Poulton ID, Hutchings C, Todryk S, Andrews L, Folgori A, Berrie E, Moyle S, Nicosia A, Colloca S, Cortese R, Siani L, Lawrie AM, Gilbert SC, Hill AVS 2012 Clinical assessment of a recombinant simian adenovirus ChAd63: a potent new vaccine vector. J Infect Dis 205:772–781. doi:10.1093/infdis/jir850. 22275401 PMC3274376

[27] Ewer KJ, O'Hara GA, Duncan CJA, Collins KA, Sheehy SH, Reyes-Sandoval A, Goodman AL, Edwards NJ, Elias SC, Halstead FD, Longley RJ, Rowland R, Poulton ID, Draper SJ, Blagborough AM, Berrie E, Moyle S, Williams N, Siani L, Folgori A, Colloca S, Sinden RE, Lawrie AM, Cortese R, Gilbert SC, Nicosia A, Hill AVS 2013 Protective CD8^+^ T-cell immunity to human malaria induced by chimpanzee adenovirus-MVA immunisation. Nat Commun 4:2836. 24284865 10.1038/ncomms3836 PMC3868203

[28] Bauza K, Malinauskas T, Pfander C, Anar B, Jones EY, Billker O, Hill AVS, Reyes-Sandoval A 2014 Efficacy of a Plasmodium vivax malaria vaccine using ChAd63 and modified vaccinia Ankara expressing thrombospondin-related anonymous protein as assessed with transgenic Plasmodium berghei parasites. Infect Immun 82:1277–1286. doi:10.1128/IAI.01187-13. 24379295 PMC3957994

[29] Reyes-Sandoval A, Wyllie DH, Bauza K, Milicic A, Forbes EK, Rollier CS, Hill AVS 2011 CD8^+^ T effector memory cells protect against liver-stage malaria. J Immunol 187:1347–1357. doi:10.4049/jimmunol.1100302. 21715686 PMC4568294

[30] Nussenzweig R, Herman R, Vanderberg J, Yoeli M, Most H 1966 Studies on sporozoite-induced infections of rodent malaria. 3. The course of sporozoite-induced Plasmodium berghei in different hosts. Am J Trop Med Hyg 15:684–689. 5917626 10.4269/ajtmh.1966.15.684

[31] Tewari R, Spaccapelo R, Bistoni F, Holder AA, Crisanti A 2002 Function of region I and II adhesive motifs of Plasmodium falciparum circumsporozoite protein in sporozoite motility and infectivity. J Biol Chem 277:47613–47618. doi:10.1074/jbc.M208453200. 12244064

[32] Potocnjak P, Yoshida N, Nussenzweig RS, Nussenzweig V 1980 Monovalent fragments (Fab) of monoclonal antibodies to a sporozoite surface antigen (Pb44) protect mice against malarial infection. J Exp Med 151:1504–1513. doi:10.1084/jem.151.6.1504. 6991628 PMC2185881

[33] Balam S, Romero JF, Bongfen SE, Guillaume P, Corradin G 2012 CSP–a model for in vivo presentation of Plasmodium berghei sporozoite antigens by hepatocytes. PLoS One 7:e51875. doi:10.1371/journal.pone.0051875. 23272182 PMC3525584

[34] Matuschewski K, Nunes AC, Nussenzweig V, Ménard R 2002 Plasmodium sporozoite invasion into insect and mammalian cells is directed by the same dual binding system. EMBO J 21:1597–1606. doi:10.1093/emboj/21.7.1597. 11927544 PMC125935

[35] Sultan AA, Thathy V, Frevert U, Robson KJ, Crisanti A, Nussenzweig V, Nussenzweig RS, Ménard R 1997 TRAP is necessary for gliding motility and infectivity of plasmodium sporozoites. Cell 90:511–522. doi:10.1016/S0092-8674(00)80511-5. 9267031

[36] Kappe S, Bruderer T, Gantt S, Fujioka H, Nussenzweig V, Ménard R 1999 Conservation of a gliding motility and cell invasion machinery in Apicomplexan parasites. J Cell Biol 147:937–944. doi:10.1083/jcb.147.5.937. 10579715 PMC2169348

[37] Longley RJ, Bauza K, Ewer KJ, Hill AVS, Spencer AJ 2015 Development of an in vitro assay and demonstration of Plasmodium berghei liver-stage inhibition by TRAP-specific CD8^+^ T cells. PLoS One 10:e0119880. doi:10.1371/journal.pone.0119880. 25822951 PMC4379172

[38] Aricescu AR, Lu W, Jones EY 2006 A time- and cost-efficient system for high-level protein production in mammalian cells. Acta Crystallogr D Biol Crystallogr 62:1243–1250. doi:10.1107/S0907444906029799. 17001101

[39] Ashok MS, Rangarajan PN 2002 Protective efficacy of a plasmid DNA encoding Japanese encephalitis virus envelope protein fused to tissue plasminogen activator signal sequences: studies in a murine intracerebral virus challenge model. Vaccine 20:1563–1570. doi:10.1016/S0264-410X(01)00492-3. 11858863

[40] Biswas S, Dicks MDJ, Long CA, Remarque EJ, Siani L, Colloca S, Cottingham MG, Holder AA, Gilbert SC, Hill AVS, Draper SJ 2011 Transgene optimization, immunogenicity and in vitro efficacy of viral vectored vaccines expressing two alleles of Plasmodium falciparum AMA1. PLoS One 6:e20977. doi:10.1371/journal.pone.0020977. 21698193 PMC3116848

[41] Draper SJ, Moore AC, Goodman AL, Long CA, Holder AA, Gilbert SC, Hill F, Hill AVS 2008 Effective induction of high-titer antibodies by viral vector vaccines. Nat Med 14:819–821. doi:10.1038/nm.1850. 18660818 PMC4822545

[42] Moore AC, Gallimore A, Draper SJ, Watkins KR, Gilbert SC, Hill AVS 2005 Anti-CD25 antibody enhancement of vaccine-induced immunogenicity: increased durable cellular immunity with reduced immunodominance. J Immunol 175:7264–7273. doi:10.4049/jimmunol.175.11.7264. 16301631

[43] Draper SJ, Goodman AL, Biswas S, Forbes EK, Moore AC, Gilbert SC, Hill AVS 2009 Recombinant viral vaccines expressing merozoite surface protein-1 induce antibody- and T cell-mediated multistage protection against malaria. Cell Host Microbe 5:95–105. doi:10.1016/j.chom.2008.12.004. 19154991 PMC2663714

[44] Van Rooijen N, Sanders A 1996 Kupffer cell depletion by liposome-delivered drugs: comparative activity of intracellular clodronate, propamidine, and ethylenediaminetetraacetic acid. Hepatology 23:1239–1243. doi:10.1002/hep.510230544. 8621159

[45] Doolan DL, Hoffman SL 2000 The complexity of protective immunity against liver-stage malaria. J Immunol 165:1453–1462. doi:10.4049/jimmunol.165.3.1453. 10903750

[46] Janse CJ, Ramesar J, Waters AP 2006 High-efficiency transfection and drug selection of genetically transformed blood stages of the rodent malaria parasite Plasmodium berghei. Nat Protoc 1:346–356. doi:10.1038/nprot.2006.53. 17406255

[47] Bejon P, Andrews L, Andersen RF, Dunachie S, Webster D, Walther M, Gilbert SC, Peto T, Hill AVS 2005 Calculation of liver-to-blood inocula, parasite growth rates, and preerythrocytic vaccine efficacy, from serial quantitative polymerase chain reaction studies of volunteers challenged with malaria sporozoites. J Infect Dis 191:619–626. doi:10.1086/427243. 15655787

[48] Ploemen IHJ, Prudêncio M, Douradinha BG, Ramesar J, Fonager J, van Gemert G-J, Luty AJF, Hermsen CC, Sauerwein RW, Baptista FG, Mota MM, Waters AP, Que I, Lowik CWGM, Khan SM, Janse CJ, Franke-Fayard BMD 2009 Visualisation and quantitative analysis of the rodent malaria liver stage by real-time imaging. PLoS One 4:e7881. doi:10.1371/journal.pone.0007881. 19924309 PMC2775639

[49] Sedegah M, Jones TR, Kaur M, Hedstrom R, Hobart P, Tine JA, Hoffman SL 1998 Boosting with recombinant vaccinia increases immunogenicity and protective efficacy of malaria DNA vaccine. Proc Natl Acad Sci U S A 95:7648–7653. doi:10.1073/pnas.95.13.7648. 9636204 PMC22711

[50] Bongfen SE, Torgler R, Romero JF, Renia L, Corradin G 2007 Plasmodium berghei-infected primary hepatocytes process and present the circumsporozoite protein to specific CD8^+^ T cells in vitro. J Immunol 178:7054–7063. doi:10.4049/jimmunol.178.11.7054. 17513754

[51] Kester KE, Cummings JF, Ofori-Anyinam O, Ockenhouse CF, Krzych U, Moris P, Schwenk R, Nielsen RA, Debebe Z, Pinelis E, Juompan L, Williams J, Dowler M, Stewart VA, Wirtz RA, Dubois M-C, Lievens M, Cohen J, Ballou WR, Heppner DG, Vaccine Evaluation Group RTSS. 2009 Randomized, double-blind, phase 2a trial of falciparum malaria vaccines RTS,S/AS01B and RTS,S/AS02A in malaria-naive adults: safety, efficacy, and immunologic associates of protection. J Infect Dis 200:337–346. doi:10.1086/600120. 19569965

[52] Draper SJ, Biswas S, Spencer AJ, Remarque EJ, Capone S, Naddeo M, Dicks MDJ, Faber BW, de Cassan SC, Folgori A, Nicosia A, Gilbert SC, Hill AVS 2010 Enhancing blood-stage malaria subunit vaccine immunogenicity in rhesus macaques by combining adenovirus, poxvirus, and protein-in-adjuvant vaccines. J Immunol 185:7583–7595. doi:10.4049/jimmunol.1001760. 21098232

[53] Sheehy SH, Duncan CJA, Elias SC, Biswas S, Collins KA, O'Hara GA, Halstead FD, Ewer KJ, Mahungu T, Spencer AJ, Miura K, Poulton ID, Dicks MDJ, Edwards NJ, Berrie E, Moyle S, Colloca S, Cortese R, Gantlett K, Long CA, Lawrie AM, Gilbert SC, Doherty T, Nicosia A, Hill AVS, Draper SJ 2012 Phase Ia clinical evaluation of the safety and immunogenicity of the Plasmodium falciparum blood-stage antigen AMA1 in ChAd63 and MVA vaccine vectors. PLoS One 7:e31208. doi:10.1371/journal.pone.0031208. 22363582 PMC3283618

[54] Sheehy SH, Duncan CJA, Elias SC, Collins KA, Ewer KJ, Spencer AJ, Williams AR, Halstead FD, Moretz SE, Miura K, Epp C, Dicks MDJ, Poulton ID, Lawrie AM, Berrie E, Moyle S, Long CA, Colloca S, Cortese R, Gilbert SC, Nicosia A, Hill AVS, Draper SJ 2011 Phase Ia clinical evaluation of the Plasmodium falciparum blood-stage antigen MSP1 in ChAd63 and MVA vaccine vectors. Mol Ther 19:2269–2276. doi:10.1038/mt.2011.176. 21862998 PMC3242658

[55] Schneider J, Gilbert SC, Blanchard TJ, Hanke T, Robson KJ, Hannan CM, Becker M, Sinden R, Smith GL, Hill AV 1998 Enhanced immunogenicity for CD8^+^ T cell induction and complete protective efficacy of malaria DNA vaccination by boosting with modified vaccinia virus Ankara. Nat Med 4:397–402. doi:10.1038/nm0498-397. 9546783

[56] Reyes-Sandoval A, Sridhar S, Berthoud T, Moore AC, Harty JT, Gilbert SC, Gao G, Ertl HCJ, Wilson JC, Hill AVS 2008 Single-dose immunogenicity and protective efficacy of simian adenoviral vectors against Plasmodium berghei. Eur J Immunol 38:732–741. doi:10.1002/eji.200737672. 18266272

[57] Reyes-Sandoval A, Berthoud T, Alder N, Siani L, Gilbert SC, Nicosia A, Colloca S, Cortese R, Hill AVS 2010 Prime-boost immunization with adenoviral and modified vaccinia virus Ankara vectors enhances the durability and polyfunctionality of protective malaria CD8^+^ T-cell responses. Infect Immun 78:145–153. doi:10.1128/IAI.00740-09. 19858306 PMC2798185

[58] Douglas AD, de Cassan SC, Dicks MDJ, Gilbert SC, Hill AVS, Draper SJ 2010 Tailoring subunit vaccine immunogenicity: maximizing antibody and T cell responses by using combinations of adenovirus, poxvirus and protein-adjuvant vaccines against Plasmodium falciparum MSP1. Vaccine 28:7167–7178. doi:10.1016/j.vaccine.2010.08.068. 20937436 PMC3404461

[59] de Cassan SC, Forbes EK, Douglas AD, Milicic A, Singh B, Gupta P, Chauhan VS, Chitnis CE, Gilbert SC, Hill AVS, Draper SJ 2011 The requirement for potent adjuvants to enhance the immunogenicity and protective efficacy of protein vaccines can be overcome by prior immunization with a recombinant adenovirus. J Immunol 187:2602–2616. doi:10.4049/jimmunol.1101004. 21813775 PMC3160495

[60] Forbes EK, Biswas S, Collins KA, Gilbert SC, Hill AVS, Draper SJ 2011 Combining liver- and blood-stage malaria viral-vectored vaccines: investigating mechanisms of CD8^+^ T cell interference. J Immunol 187:3738–3750. doi:10.4049/jimmunol.1003783. 21876036 PMC3284248

[61] Ockenhouse CF, Sun P-F, Lanar DE, Wellde BT, Hall BT, Kester K, Stoute JA, Magill A, Krzych U, Farley L, Wirtz RA, Sadoff JC, Kaslow DC, Kumar S, Church LWP, Crutcher JM, Wizel B, Hoffman S, Lalvani A, Hill AVS, Tine JA, Guito KP, de Taisne C, Anders R, Horii T, Paoletti E, Ballou WR 1998 Phase I/IIa safety, immunogenicity, and efficacy trial of NYVAC-Pf7, a pox-vectored, multiantigen, multistage vaccine candidate for Plasmodium falciparum malaria. J Infect Dis 177:1664–1673. doi:10.1086/515331. 9607847

[62] Richie TL, Charoenvit Y, Wang R, Epstein JE, Hedstrom RC, Kumar S, Luke TC, Freilich DA, Aguiar JC, Sacci JB, Sedegah M, Nosek RA, la Vega de P, Berzins MP, Majam VF, Abot EN, Ganeshan H, Richie NO, Banania JG, Baraceros MFB, Geter TG, Mere R, Bebris L, Limbach K, Hickey BW, Lanar DE, Ng J, Shi M, Hobart PM, Norman JA, Soisson LA, Hollingdale MR, Rogers WO, Doolan DL, Hoffman SL 2012 Clinical trial in healthy malaria-naïve adults to evaluate the safety, tolerability, immunogenicity and efficacy of MuStDO5, a five-gene, sporozoite/hepatic stage Plasmodium falciparum DNA vaccine combined with escalating dose human GM-CSF DNA. Hum Vaccin Immunother 8:1564–1584. doi:10.4161/hv.22129. 23151451 PMC3601132

[63] Doolan DL, Martinez-Alier N 2006 Immune response to pre-erythrocytic stages of malaria parasites. Curr Mol Med 6:169–185. doi:10.2174/156652406776055249. 16515509

[64] Slifka MK, Pagarigan RR, Whitton JL 2000 NK markers are expressed on a high percentage of virus-specific CD8^+^ and CD4^+^ T cells. J Immunol 164:2009–2015. doi:10.4049/jimmunol.164.4.2009. 10657652

[65] Nishikado H, Mukai K, Kawano Y, Minegishi Y, Karasuyama H 2011 NK cell-depleting anti-asialo GM1 antibody exhibits a lethal off-target effect on basophils in vivo. J Immunol 186:5766–5771. doi:10.4049/jimmunol.1100370. 21490162

[66] Cerami C, Frevert U, Sinnis P, Takacs B, Clavijo P, Santos MJ, Nussenzweig V 1992 The basolateral domain of the hepatocyte plasma membrane bears receptors for the circumsporozoite protein of Plasmodium falciparum sporozoites. Cell 70:1021–1033. doi:10.1016/0092-8674(92)90251-7. 1326407

[67] Touray MG, Seeley DC, Miller LH 1994 Plasmodium gallinaceum: differential lysis of two developmental stages of malaria sporozoites by the alternative pathway of complement. Exp Parasitol 78:294–301. doi:10.1006/expr.1994.1031. 8162961

[68] Schwenk R, Asher LV, Chalom I, Lanar D, Sun P, White K, Keil D, Kester KE, Stoute J, Heppner DG, Krzych U 2003 Opsonization by antigen-specific antibodies as a mechanism of protective immunity induced by Plasmodium falciparum circumsporozoite protein-based vaccine. Parasite Immunol 25:17–25. doi:10.1046/j.1365-3024.2003.00495.x. 12753434

[69] Doolan DL, Hoffman SL 1999 IL-12 and NK cells are required for antigen-specific adaptive immunity against malaria initiated by CD8^+^ T cells in the Plasmodium yoelii model. J Immunol 163:884–892. 10395683

[70] Horowitz A, Hafalla JCR, King E, Lusingu J, Dekker D, Leach A, Moris P, Cohen J, Vekemans J, Villafana T, Corran PH, Bejon P, Drakeley CJ, Seidlein von L, Riley EM 2012 Antigen-specific IL-2 secretion correlates with NK cell responses after immunization of Tanzanian children with the RTS,S/AS01 malaria vaccine. J Immunol 188:5054–5062. doi:10.4049/jimmunol.1102710. 22504653 PMC3378032

[71] McCall MBB, Roestenberg M, Ploemen I, Teirlinck A, Hopman J, de Mast Q, Dolo A, Doumbo OK, Luty A, van der Ven AJAM, Hermsen CC, Sauerwein RW 2010 Memory-like IFN-γ response by NK cells following malaria infection reveals the crucial role of T cells in NK cell activation by Plasmodium falciparum. Eur J Immunol 40:3472–3477. doi:10.1002/eji.201040587. 21072880

[72] Rodrigues MM, Cordey AS, Arreaza G, Corradin G, Romero P, Maryanski JL, Nussenzweig RS, Zavala F 1991 CD8^+^ cytolytic T cell clones derived against the Plasmodium yoelii circumsporozoite protein protect against malaria. Int Immunol 3:579–585. doi:10.1093/intimm/3.6.579. 1716146

